# Recurrent Significant Bleeding From Abdominal Wall Giant Pyogenic Granuloma Requiring Multiple Blood Transfusions

**DOI:** 10.7759/cureus.28863

**Published:** 2022-09-06

**Authors:** Mohamed Ahmed, Maya Itani, Ramsey Elsayed, Asef Bawahab, Yusef J Buti

**Affiliations:** 1 Surgery, University of California, Riverside, USA; 2 Surgery, Medical University of the Americas, Corona, USA; 3 Surgery, Temecula Valley Hospital, Universal Health Services, SoCal MEC (Southern California Medical Education Consortium), Temecula, USA; 4 General Surgery, Universal Health Services, SoCal MEC (Southern California Medical Education Consortium), Temecula, USA; 5 Surgery, Universal Health Services, SoCal MEC (Southern California Medical Education Consortium), Temecula, USA

**Keywords:** benign or malignant skin conditions, indolent skin lesion, bleeding risk, severe anaemia, pyogenic granuloma

## Abstract

Pyogenic granuloma (PG), also known as lobular capillary hemangioma, is a common benign vascular proliferation of unclear etiology. The proposed etiology includes trauma, infection, and preceding dermatoses. All age groups and both sexes can be affected. It should be differentiated from malignant tumors such as amelanotic melanoma, basal cell carcinoma, and spindle cell tumor. We present a case of recurrent significant bleeding from the abdominal wall mass requiring blood transfusion. Surgical excision is the recommended treatment. The aim of this report is to shed the light on this rare presentation.

## Introduction

Inflammatory hyperplasia of mucosa or the skin results in a benign vascular neoplasm known as pyogenic granuloma (PG). The name is misleading as it is not a true granuloma nor is pus seen histologically. The exact etiology is unknown and has been proposed to be a response to various stimuli such as chronic irritation, trauma, hormones, and drugs [1]. Size can be up to many centimeters, usually, and none is tender to touch, sessile, or pedunculated [2]. Histologically granulation tissue, chronic inflammatory infiltrate, and marked vascular proliferation with fibrin cover the ulcerated PG, and fibrosis is seen in older lesions [3]. The clinical differential diagnosis includes peripheral giant cell granuloma, peripheral ossifying fibroma, metastases of malignant tumors, hemangioma, conventional granulation tissue, fistula, inflammatory gingival hyperplasia, Kaposi's sarcoma, angiosarcoma, non-Hodgkin's lymphoma, and cutaneous horn in the lower lip, and hence a biopsy is required for the diagnosis of PG [4]. Surgical excision is the most common treatment with a 3% recurrence rate; however, cryotherapy and laser surgery can also be used [5]. Electrodesiccation injection of absolute ethanol has also been used [6].

## Case presentation

**Figure 1:**
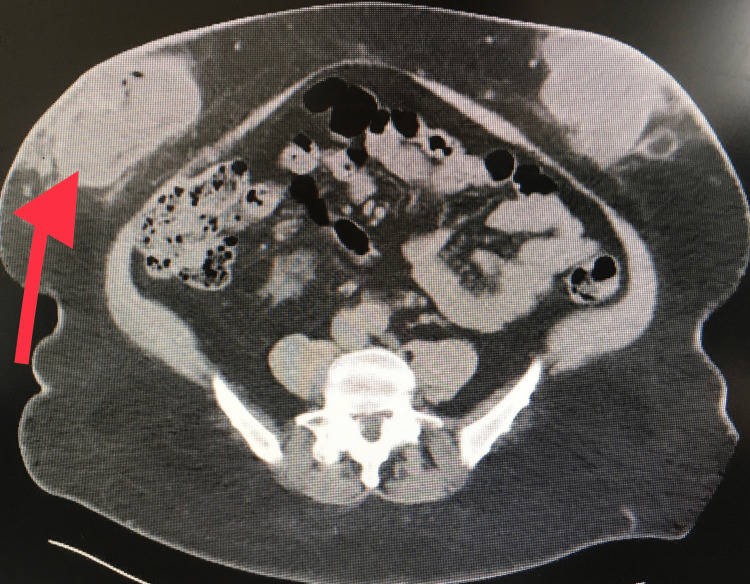
CT of the abdomen Rim-enhancing right lower quadrant mass concerning for abscess or hematoma (red arrow).

A 68-year-old African American female presented to our emergency room with recurrent profuse bleeding abdominal wall mass. The mass was noticed a year ago and has been slowly increasing in size and ulcerating. The patient believed that it is at the site of her subcutaneous Lovenox injection. She was admitted one month prior to presentation with acute blood loss anemia, hemoglobin of 7 g/dL (reference: 12-16 g/dL), and CT finding consistent with a rim-enhancing lesion suspicious of an abscess or hematoma (Figure 1).

**Figure 2:**
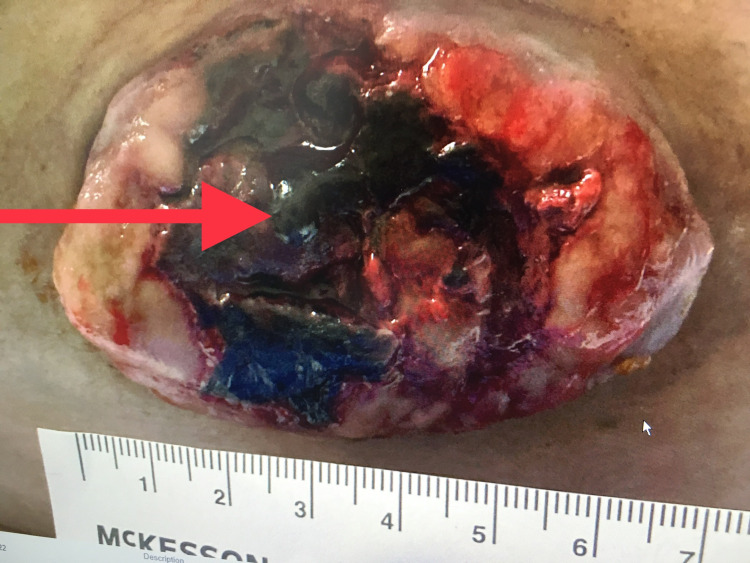
Right lower quadrant abdominal wall mass. Biopsy-proven pyogenic granuloma. Active bleeding is controlled by applying pressure. Blood clot (red arrow).

Bleeding was controlled with topical hemostatic agents and the application of pressure. Biopsy of the lesion performed during her earlier admission was consistent with PG. On admission, her laboratory findings revealed hemoglobin of 8.5 g/dL (reference: 12-16 g/dL), INR of 1.05 (reference: 0.91 - 1.116), and PTT of 58.4 seconds (reference: 23.9-31.3 seconds). The patient's past medical history includes obesity, hypertension, non-insulin-dependent diabetes, obstructive sleep apnea, deep venous thrombosis, and pulmonary embolism for which, she receives twice daily Lovenox (enoxaparin sodium) 120mg subcutaneous. The bleeding was controlled with manual pressure (Figure 2).

**Figure 3:**
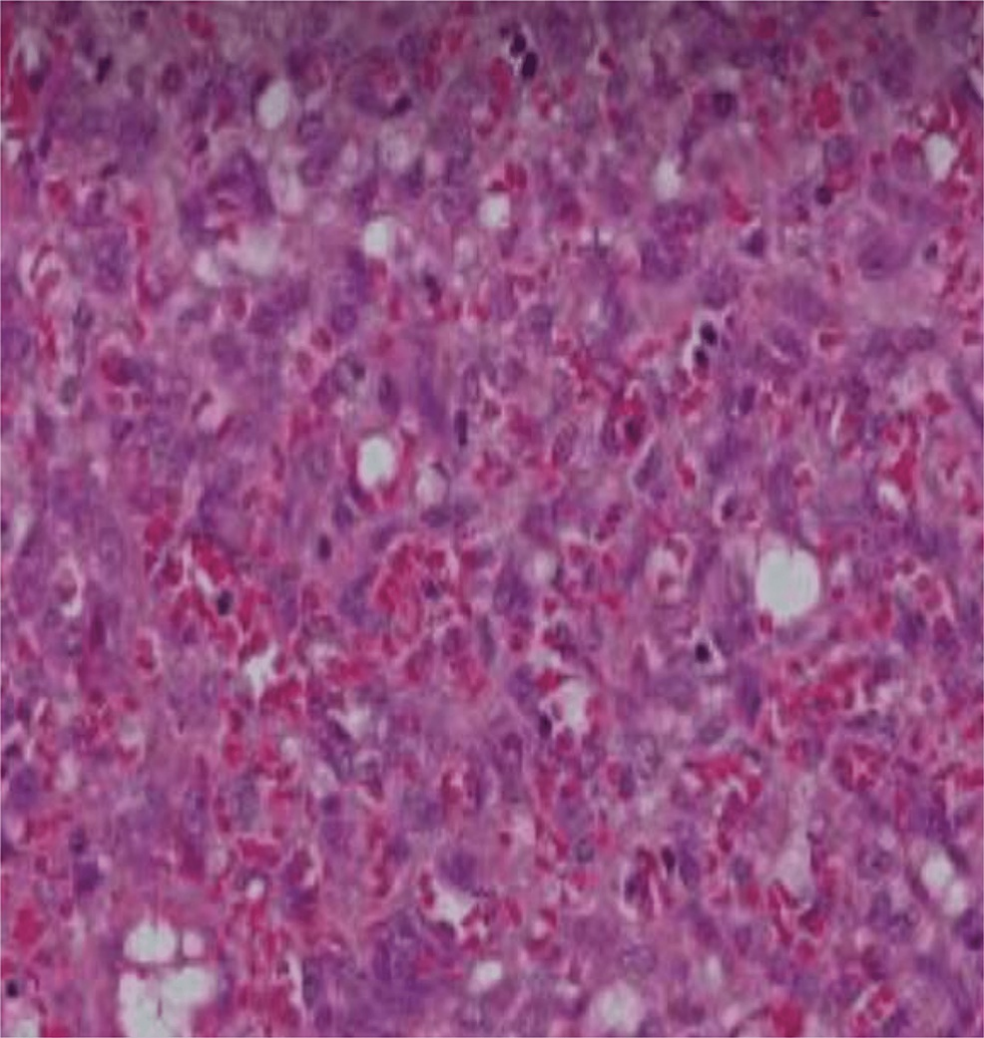
Histopathology Benign pyogenic granuloma with reactive changes.

Surgical resection was performed with an elliptical incision facilitating primary closure with drain placement. Histopathology was consistent with benign PG (Figure 3).

The patient did well. Anticoagulation was resumed on postoperative day 1 and she was discharged from the hospital on postoperative day 2. Drain placed during surgery was removed during clinic follow-up.

## Discussion

PG is a bright red-brown benign tumor with a thin intact epidermis usually presenting as a rapidly growing mostly painless red papule or nodule with a propensity to bleed spontaneously or with manipulation and results from an inflammatory reaction and often occurs in the mouth or on the hands or feet, though it may occur on the upper back or neck as well [7]. Reports on abdominal wall PG are scarce in the literature.

PG is a misnomer unrelated to infection, does not contain pus nor occurs as a consequence of granulomatous inflammation, and is predominantly seen in young female gingiva [8]. As a result of hormonal imbalance, gingiva PG has been reported to occur in up to 5% of pregnant women during the second and third trimesters and has been referred to as pregnancy tumor or granuloma gravidarum [9].

The etiology remains unknown and is proposed to be multifactorial including trauma, underlying microscopic arteriovenous malformations, angiogenic factors production, cytogenetic abnormalities, hormonal influences, and viral oncogenes. At a molecular level, overexpression of transcription factors P-ATF2 (regulate gene expression in response to environmental changes) and STAT3 (signal transducer and activator of transcription) was also implicated to play a role [10]. Minor trauma or underlying cutaneous disease leading to excessive local production of tumor angiogenesis factor might be an important factor in the pathogenesis of PG [11].

Differential diagnoses include malignant tumors, such as nodular-type basal cell carcinoma, squamous cell carcinoma, and melanoma. PG can imitate amelanotic melanoma clinically, and histopathological evaluation is required in order to differentiate the two pathologies [12].

The diagnosis of PG is often straightforward, but a biopsy is necessary for differential diagnoses including malignant tumors. Histologically, the presence of numerous endothelium-lined vascular spaces and the proliferation of fibroblast cells are characteristic features of PG [13].

Treatment is required to confirm the diagnosis, associated risk of ulceration and bleeding, and cosmetic concerns. Excisional surgery, topical medical therapy with silver nitrate, phenol, podophyllin, imiquimod, and beta-blockers, cryotherapy, electrocautery, and laser ablation have been used as treatment options [14].

The use of neodymium-doped yttrium aluminum garnet (Nd:YAG laser) is associated with a lower risk of bleeding but a high recurrence rate in granuloma gravidorum [15].

To avoid any recurrence, a full-thickness skin excision and linear closure are recommended, and a 43.5% recurrence rate has been reported in lesions treated by shave (intradermal) excision and cautery or cautery alone [16].

## Conclusions

PG is a rapidly growing vascular papule or polyp. It bleeds easily after minor trauma. It can occur in the skin or mucosa. The diagnosis of PG is often straightforward, but a biopsy is necessary for differential diagnoses including malignant tumors. Management includes cryotherapy, electrocoagulation, phenol or alcohol injection, topical beta-blocker, and excision. Full-thickness surgical excision is the preferred treatment with no reported recurrence.
